# Development of a validation imaging dataset for Molecular Radiotherapy dosimetry multicenter intercomparison exercises based on anthropomorphic phantoms

**DOI:** 10.1016/j.ejmp.2023.102583

**Published:** 2023-05

**Authors:** Andrew P. Robinson, Nick Calvert, Jill Tipping, Ana M. Denis-Bacelar, Kelley M. Ferreira, Michael Lassmann, Johannes Tran-Gia, The MRTDosimetry Collaboration

**Affiliations:** a National Physical Laboratory, Teddington, TW11 0LW, United Kingdom; b Christie Medical Physics and Engineering (CMPE), The Christie NHS Foundation Trust, Wilmslow Road, Manchester M20 4BX, United Kingdom; c Schuster Laboratory, School of Physics and Astronomy, The University of Manchester, Manchester M13 9PL, United Kingdom; d Department of Nuclear Medicine, University Hospital Würzburg, Oberdürrbacher Str. 6, 97080 Würzburg, Germany

**Keywords:** MRT dosimetry, Phantom, Lu-177, Validation

## Abstract

Validation of a Molecular Radiotherapy (MRT) dosimetry system requires imaging data for which an accompanying “ground truth” pharmacokinetic model and absorbed dose calculation are known.

**Methods:**

We present a methodology for production of a validation dataset for image based ^177^Lu dotatate dosimetry calculations. A pharmacokinetic model is presented with activity concentrations corresponding to common imaging timepoints. Anthropomorphic 3D printed phantoms, corresponding to the organs at risk, have been developed to provide SPECT/CT and Whole Body imaging with known organ activities corresponding to common clinical timepoints.

**Results:**

Results for the accuracy of phantom filling reproduce the activity concentrations from the pharmacokinetic model for all timepoints and organs within measurement uncertainties, with a mean deviation of 0.6(8)%. The imaging dataset, ancillary data and phantoms designs are provided as a source of well characterized input data for the validation of clinical MRT dosimetry systems.

**Conclusions:**

The combination of pharmacokinetic modelling with the use of anthropomorphic 3D printed phantoms are a promising procedure to provide data for the validation of Molecular Radiotherapy Dosimetry systems, allowing multicentre comparisons.

## Highlights

• An imaging dataset for validation of a Molecular Radiotherapy dosimetry system has been produced.
• A method for producing a dataset representing a ground truth PK model is presented.
• PK model reproduced all time points and organs within measurement uncertainties.
• Dataset is available allowing institutions to compare calculated dosimetry values.

## 1 Introduction

Absolute validation of a Molecular Radiotherapy (MRT) dosimetry system is a seemingly ambitious task. MRT dosimetry relies on a complex chain of operations that are dependent on each other and contribute to the overall accuracy and precision of the final dosimetry calculation. There are many choices involved in a dosimetry calculation, from the techniques used to define volumes of interest, to the fitting of time activity curves and choice of dose S-factors or kernels, where each choice will affect the outcome. Dosimetry comparison exercises of different systems based on patient data highlight relative differences, but are weakened by the fact that when using patient data, the inherent ground truth is unknown [1], [2]. A recent overview of commercial treatment planning systems (TPS) for MRT recognized the need to assess uncertainty in the dosimetric process and noted that the impact on TPS results still needs to be assessed [3].

This study was performed during the EMPIR MRTDosimetry project. The objective of the MRTDosimetry project was to provide a methodology to link the chain of measurements within a dosimetry calculation in a way to be able to ascertain the accuracy and uncertainties of each step. In this way the performance of a dosimetry calculation can be understood by its key operations. The system was developed and trialed as a dosimetric cross comparison exercise for the MRTDosimetry project. A model system of data was developed to allow centres to perform dosimetry calculations on identical datasets, representing patient imaging performed post ^177^Lu -DOTATATE administration with a known absorbed dose ground truth.

The use of 3D printing has been established in a wide range of medical applications [4], [5] and has previously been used to improve the accuracy of MRT dosimetry [6], [7], [8], [9], [10], [11]. In this current work a 3D printed phantom based on the ICRP 110 female computational phantom [12] was developed to produce a patient-representative Single Photon Emission Computed Tomography (SPECT) imaging dataset. To achieve the most realistic dataset possible, multiple SPECT/CT measurements, corresponding to common timepoints for post-therapy imaging, were performed of a 3D printed organ phantom filled with radioactive solutions following a realistic biokinetic distribution for ^177^Lu-DOTATATE Peptide Receptor Radionuclide Therapy (PRRT) [13]. SPECT with sequential CT and Whole Body (WB) imaging was performed using a GE Discovery 670 SPECT/CT camera.

This work describes the production of a validation imaging dataset for Molecular Radiotherapy Dosimetry Multicenter Intercomparison Exercises, based on a novel 3D printed phantom. The design and production of the phantom, its filling, and the SPECT imaging and image calibration is presented. The resulting 3D models, pharmacokinetic curves, SPECT/CT and WB images have been made available online as a standard reference database for validation of MRT dosimetry calculations [14].

**Fig. 1:**
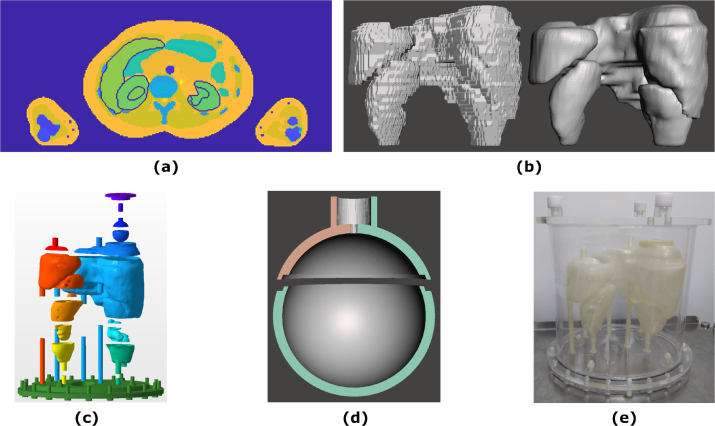
**(a)** A slice through the ICRP110 Female Computational Phantom. The liver and kidneys are outlined. **(b)** The Stereolitography (STL) files of Organs At Risk before and after smoothing. **(c)** An exploded view of the phantom design. **(d)** A cross-section through the spherical tumour showing the join between the separate parts. **(e)** A photograph of the assembled phantom.

## 2 Materials and methods

### 2.1 Fabrication of the anthropomorphic phantom

The ICRP110 Female Anthropomorphic Phantom [12] was used as the basis for the anthropomorphic phantom and the kidneys, liver, and spleen were chosen as the Organs At Risk (OARs). The ICRP 110 voxelized phantom is a well defined reference standard phantom used for dosimetry. In addition there are several studies of Monte Carlo simulations of this phantom to determine dose conversion factors for dosimetry. Dose conversion coefficients for the ICRP110 voxel phantom have been calculated with the Geant4 Monte Carlo code [15]. Fig. 1(a) shows a cross-sectional slice through the computational phantom with the kidneys and liver outlined. The phantom data were imported into MATLAB [16], and the OARs were converted from the computational phantom to a set of triangular meshes, Laplacian smoothing was applied to the meshes to remove the voxelization, and the meshes were exported in STL format. Fig. 1(b) shows the STL format data before and after smoothing. In the ICRP110 phantom the kidneys consist of three compartments: cortex, medulla, and renal pelvis. For the 3D printed phantom the medulla and renal pelvis were combined to create kidney models with two compartments: cortex and medulla + renal pelvis (medulla). Some voxels in the ICRP110 phantom were manually changed between medulla and cortex to remove any discontinuities or self-intersecting regions in the resulting meshes. The STL files were imported into Autodesk Meshmixer [17] and extruded outwards by 2mm (1mm for the medulla compartments) to create a set of hollow shells. The right and left kidney were translated to accommodate the extruded shells and to ensure they did not overlap with the liver and spleen, respectively. The right kidney was then joined to the liver and the left kidney was joined to the spleen to ensure a constant relative position and orientation when imaging, see Fig. 1(c). A 15.9 mL spherical insert (tumour) was added to the liver model as a separate fillable compartment to represent uptake in a tumour.

The shells were designed in parts to ensure they could be printed and the support material could be easily removed. An interlocking join was designed into each part so the parts could only fit together in one orientation and to reduce the likelihood of leaking after printing and sealing. A cross-sectional view of the spherical insert with a join is shown in Fig. 1(d). Filling and support ports were designed into the surfaces so that components could be easily filled and emptied, and could be supported using a set of support rods. A large cap was added to the top of the liver to provide easy filling and emptying, and also allow the addition of the spherical tumour in the liver. The tumour was designed with 2.0mm thick walls and a single needle filling port to reduce the likelihood of air bubbles when filling. All the boolean operations applied to the STL files, to create the filling and support ports and the joins, were performed in Autodesk Netfabb [18]. An exploded view of the final phantom design is given in Fig. 1(c).

The parts were printed using an in-house Ultimaker 3 Extended using polylactic acid (PLA), with a layer height of 0.1mm and an in-fill of 100%. The tissue equivalence of PLA at photon energies appropriate for quantitative imaging in radionuclide therapy was been demonstrated [19] and shown to compare favourably with Perspex© commonly used in commercial phantoms [20]. Multiple copies of the phantom were printed to avoid the need to empty the phantom between fillings. After removing the support material the separate parts were joined together using a small amount of Chloroform (CHCl_3_) applied to each side of the join. The parts were leak tested before a thin layer of Araldite® 2020 epoxy was applied to the outside to reduce the likelihood of leaks developing over time. Rather than directly tapping the filling and support holes in the printed part, small nylon inserts were manufactured that were tapped and then glued into the holes to ensure the printed parts did not get damaged in the tapping process. The filling holes were sealed with a nylon M6 bolt, with the exception of the medullas which were sealed with nylon M5 bolts. A rubber gasket was used to prevent any leakage from the liver cap. A baseplate to hold the support rods for the printed inserts in place was designed and laser cut out of Acrylic at the University of Manchester. To complete the phantom a large elliptical phantom, with internally major and minor axes of 258mm and 198mm, respectively, and a height of 268mm, was constructed from Perspex© at the Christie NHS Foundation Trust mechanical workshop. The baseplate was designed to attach to the base of the elliptical housing. The elliptical housing could then be filled with an activity, to mimic background activity in the adjacent tissues. An elliptical housing was chosen (rather than the voxelized model) to simplify construction and removal of the organ inserts. A photograph of the assembled phantom within the elliptical housing is shown in Fig. 1(e).

### 2.2 Two organ phantom model

**Fig. 2:**
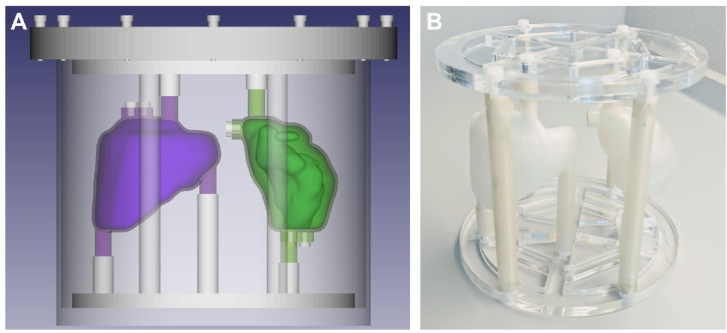
**(a)** Design of the spleen and kidney phantom including mounting plate for cylindrical Jaszczak phantom. **(b)** Photograph showing the assembled phantom in the support frame.

To facilitate the multi-centre evaluation of quantitative ^177^Lu SPECT/CT imaging performed within the MRTDosimetry project [21] a simplified phantom insert containing only the spleen and right kidney compartments was designed. The same production approach as detailed for the larger phantom was employed to create an insert for a standard cylindrical Jaszczak phantom (nominal volume 6.9 L) [22] designed to be attached using laser-cut mounting plates and support rods (see Fig. 2).

**Fig. 3:**
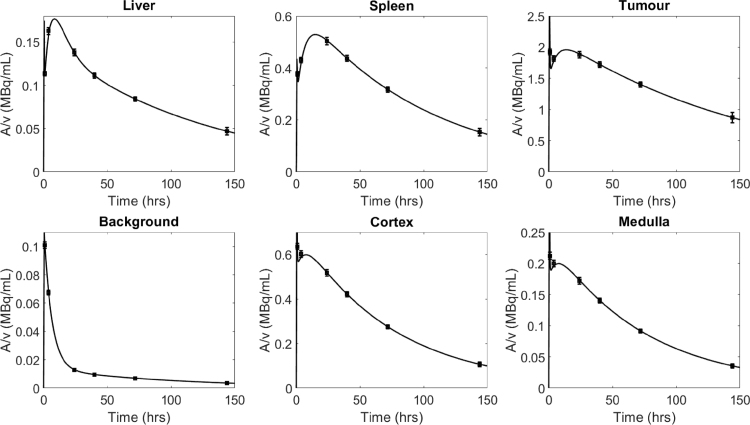
The dispensed activity concentration (black squares) of the compartments at each SPECT imaging time point (body contour enabled) compared to the curves generated by the pharmacokinetic model (black line). A common stock solution was used for the left and right kidney compartments.

### 2.3 Pharmacokinetic model

The activity concentration to be filled in each compartment was based on the pharmacokinetic model developed in [13]. The model was implemented in the SAAM II software for kinetic analysis [23], with the compartment volumes adjusted to match the STL volumes. The model was then solved in MATLAB using the Fourth-Order Runge–Kutta (RK4) algorithm, with a step size of ΔT=1 min, to generate the activity concentration in each compartment as a function of time (T) up to 100 days post injection, assuming an administered activity of 7.4 GBq. For simplicity, the kidney medulla activity concentration was set to be one third of the activity concentration in the kidney cortex and the activity concentration in the elliptical phantom background matched that of the rest of body in the pharmacokinetic model. The modelled activity concentration in each compartment as a function of time, up to T=150 h post injection, is shown in Fig. 3 (lines), the data has been corrected to take into account the radioactive decay of ^177^Lu [24].

### 2.4 Dataset production

#### 2.4.1 Phantom preparation

The phantom was filled six times with activity concentrations corresponding to different time points from the pharmacokinetic model (see Fig. 3). The time points chosen were T=1 h, 4 h, 24 h, 40 h, 72 h and 144 h, a combination of the imaging times used by two clinical members of the MRTDosimetry consortium and commonly used by a number of clinical centres. A vial of ^177^LuCl was diluted with 0.04M HCl to 4 mL in a P6 vial and the activity was measured on a Capintec CRC-55tR radionuclide calibrator using a factor linked to the UK National Physical Laboratory (NPL) primary standard of activity of ^177^Lu. This activity value was decay corrected to the estimated initial SPECT acquisition start time to ensure that the activity concentration at scanning time would match that calculated by the model. The activity required for all of the compartments, plus an extra 15%, was drawn up and dispensed into an Erlenmeyer flask and further diluted to 100 mL with 0.04M HCl. A beaker of active solution was prepared for each compartment (liver, spleen, cortex, medulla and tumour) by drawing up 110% of the activity required for each compartment and diluting to approximately 105% of the compartment volume. Each beaker was then further diluted with 0.04 M HCl until the volume of the solution was correct to within 0.01 mL to ensure the activity concentration at the time of imaging matched the model. Each phantom insert was then weighed empty before the solution from the appropriate beaker was dispensed using a syringe. The weight of the organ was checked as it reached the end of filling and careful adjustments were made to ensure that as many air bubbles as possible were removed before the final filling. The insert was sealed with a nylon bolt and rubber o-ring and weighed when full to calculate the total volume of solution dispensed, subsequently used to calculate the final activity in each component. The filled inserts were assembled in the elliptical phantom with the baseplate and the phantom and inserts weighed. The phantom background was half filled with 0.04M HCl before the required activity was dispensed using a syringe with a linked calibration factor. The background was then filled, sealed and weighed to get the total volume of solution in the background compartment. All weight measurements were performed using a calibrated OHAUS PA2102 balance with a readability of 0.01 g, except for the liver which was measured on a Kern KB 10k0.05N balance with a readability of 0.05 g and the fully assembled phantom which was measured empty and full using a Kern CFS 50K-3 balance with a readability of 1 g.

#### 2.4.2 SPECT and WB imaging

The phantom was filled with activity concentrations corresponding to the six different time points described previously. SPECT/CT imaging was performed on a Discovery 670 (GE Healthcare), using the acquisition parameters in Table 1, initially with body contour applied and then repeated using a fixed detector radius of 27.5 cm. Image reconstruction was performed using Hermes Medical Solutions Hybrid Reconstruction 3.0 (Stockholm, Sweden) using the standard Ordered Subset Expectation Maximization (OSEM) algorithm with 4 iterations and 10 subsets. A CT-derived attenuation correction was applied, the SPECT projections were scatter corrected using the Triple Energy Window (TEW) method [25], and resolution recovery was applied. WB imaging was also performed using a scan speed of 6 cm min^−1^ with body contour enabled and a pixel size of $2.21\times 2.21\;{\mathrm{mm}}^{2}$. All raw data sinograms were stored to allow future reconstructions using different techniques/parameters.

**Table 1:** SPECT Acquisition parameters.

Matrix Size	128 × 128
Collimator	Medium Energy General Purpose
Views per Head	60 (120 in total)
Time per View	30 s
Body Contour	Enabled
Photopeak	208.0 keV ±10%
Lower Scatter Window	181.3 keV ±3%
Upper Scatter Window	236.4 keV ±3%

#### 2.4.3 Ancillary SPECT/CT calibration data

Quantitative SPECT imaging (QI) requires a camera- and image reconstruction-specific calibration factor to be determined from a phantom measurement (typically with a sufficiently large volume to minimize partial volume effects). In addition, to account for spill-out partial volume effects, a recovery curve based partial volume correction method can be used [26], [27]. An experimental protocol detailing these approaches is presented in [21]. Comprehensive validation of a MRT dosimetry system requires accurate determination of the uncertainty on the complete measurement chain, including the QI used as input to dosimetry calculations. To this end an accompanying SPECT/CT dataset, suitable for performing QI calibration, was acquired consisting of:

• A cylindrical Jaszczak phantom (nominal volume 6.9 L) filled with 409(14) MBq of ^177^Lu diluted in HCl.
• An IEC NEMA PET body phantom [28] with uniform activity distribution in the six-sphere inserts and a water-filled background.

To prepare the NEMA phantom a stock solution of ^177^Lu diluted in 0.04M HCl was prepared with an activity concentration of 2.00(6) MBq/mL and dispensed into the hollow spheres of the phantom. Each sphere was measured empty and full to calculate the volumes, V=0.53(2) mL, 1.17(2) mL, 2.54(2) mL, 5.58(2) mL, 11.59(2) mL, 26.7(2) mL. SPECT/CT data was acquired for both phantoms using the acquisition parameters described in Table 1. This ancillary data has been made available as part of the complete dataset, discussed in Section 4, to allow validation of dosimetry systems which include QI calibration.

## 3 Results

When producing a validation dataset for MRT dosimetry calculations the accuracy of the acquired data (in comparison to the adopted pharmacokinetic model) establishes the achievable accuracy of comparisons utilizing the dataset.

### 3.1 Volume filling accuracy

The filled volume of each insert at each scan as a percentage of the STL volume is provided in Table 2, along with an estimate of the associated uncertainty. In general the filled volumes were lower than the theoretical STL volume due to the inclusion of air bubbles when filling and sealing.

**Table 2:** The STL volume and percentage of the volume filled prior to each scan (standard uncertainties referred to the corresponding last digits of the quoted result are quoted in parentheses).

Organ	STL Volume (mL)	Filling volume (% of STL volume)					
		Scan 1	Scan 2	Scan 3	Scan 4	Scan 5	Scan 6
Liver	1306.7	100.08(1)	99.84(1)	100.04(1)	99.95(1)	100.13(1)	99.89(1)
Spleen	124.9	99.43(2)	99.45(2)	99.40(2)	99.21(2)	99.29(2)	99.23(2)
Tumour	15.9	99.6(1)	99.4(1)	99.2(1)	99.5(1)	99.7(1)	94.8(1)
Left kidney cortex	90.9	97.69(2)	96.48(2)	98.92(2)	98.14(2)	98.33(2)	98.40(2)
Left kidney medulla	42.9	99.05(5)	97.19(5)	102.04(5)	97.47(5)	97.72(5)	97.19(5)
Right kidney cortex	75.7	99.29(3)	98.89(3)	99.59(3)	98.65(3)	99.48(3)	98.72(3)
Right kidney medulla	35.7	96.20(6)	97.06(6)	96.56(6)	96.45(6)	97.32(6)	97.60(6)
Background	8297	98.25(3)	98.25(3)	98.25(3)	98.35(3)	98.28(3)	98.24(3)

### 3.2 Comparison to pharmacokinetic model

The filled activity concentration in each compartment at each initial body contour enabled SPECT imaging time is plotted in Fig. 3 (black squares) along with the data generated by the model (solid line). The method of filling described previously ensured that the measured activity concentration in each compartment was in agreement with the pharmacokinetic model to within measurement uncertainties at each time point (mean deviation from model for all inserts and scans 0.6(8)%). Due to the physical decay of ^177^Lu, the activity concentrations at the time of subsequent WB and SPECT imaging without body contour were lower than those in Fig. 3 as a slight physical decay occurred between scans (mean deviation from model 0.8(7)%).

### 3.3 Phantom imaging

**Fig. 4:**
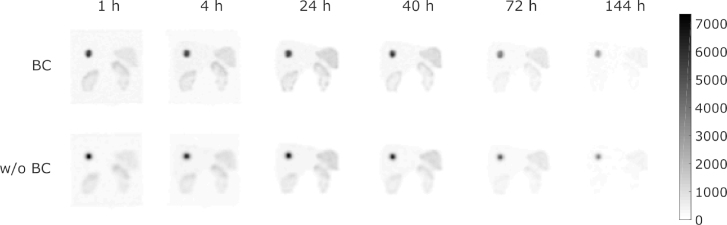
Two-dimensional MIPs of each reconstructed SPECT image acquired with (top) and without (bottom) body contour enabled.

**Fig. 5:**
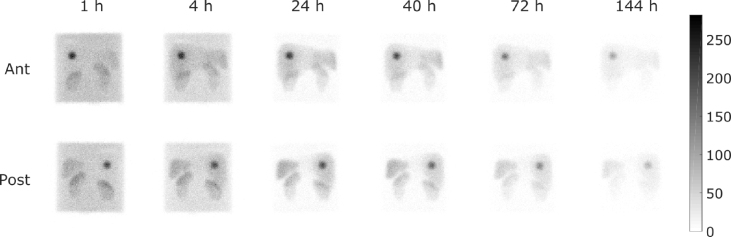
Anterior and Posterior WB images acquired at each time point with TEW scatter correction applied.

A two-dimensional Maximum Intensity Projection (MIP) of each reconstructed image, for data acquired with and without body contour enabled, is shown in Fig. 4 and the WB images in Fig. 5. All data were acquired using the acquisition settings described in Table 1, and reconstructed consistently. The original (GE compatible) DICOM format data were also converted to a Siemens compatible DICOM using PyDicom [29] to increase potential compatibility of the dataset with multiple vendor systems.

## 4 Discussion

Comprehensive phantom validation of a MRT Dosimetry platform requires accurate, well characterized, imaging data as input. Ground truth values for absorbed dose, corresponding to an appropriate pharmacokinetic model, are also required. The requirement for a ground truth relating activity quantified from imaging with absorbed dose values necessitates the use of phantom imaging or Monte Carlo simulation of an imaging system. In both cases for a meaningful comparison the validation (ground truth) data must include an assessment of uncertainties [30]. When using a phantom for quantitative studies the uncertainty of the dispensed activity is a fundamental limit to the accuracy for many studies where phantoms are used for calibration or validation. This is particularly important when using phantoms for dosimetry studies which require data at multiple time points. In addition, when considering multi-centre studies (for example [21]) traceability of activity measurements [31] to an appropriate primary standard through an NMI is essential to ensure harmonization between centres. It is also important to emphasize that when considering the use of imaging data from physical phantoms as input to validate a MRT dosimetry calculations, calibration data relating the count rate in the detector to activity must also be provided. It is essential that this data is acquired on the same camera as the phantom data, with a well established QC procedure in place, and that the reconstructed calibration images are processed with the same parameters as the phantom data. If singoram data is used as the input then any changes to the reconstruction parameters must be applied to calibration and validation datasets.

In this work the accuracy of the dispensed volumes (Table 2), showed the highest uncertainty for the tumour and medulla, with their small size and complicated shape trapping air bubbles. The reported liver filling volumes above 100% of the STL volume may have been caused by the cap not sitting in the top of the insert in the same fashion as modelled in the STL file due to the rubber o-ring. The overfill in the left kidney medulla prior to Scan 3 corresponded to 0.8 mL. The background compartment was underfilled by 1.65–1.76% which may have been caused by tolerances in the manufacturing process deviating from the initial design.

The adoption of a standard reference dataset for assessing performance and providing Quality Assurance (QA) for MRT dosimetry platforms is an important step towards harmonization and reproducibility for clinical MRT dose calculations. In this spirit of open science, the complete imaging dataset presented in this work has been made available along with the STL files required to print a copy of the phantom [14]. The ancillary SPECT/CT calibration data, the integrals of the time dependent activity curves generated by the pharmacokinetic model and Monte Carlo generated absorbed dose values for the phantom have also been provided, allowing for institutions to compare their calculated dosimetry values to the ground truth. Within the MRTDosimetry project this dataset has been utilized in a multicentre intercomparison exercise of MRT ^177^Lu PRRT dosimetry calculations. A manuscript presenting the methodology used to calculate the ground truth absorbed dose distribution and analysis of dosimetric data from the intercomparison exercise is currently in preparation. The dataset has been designed to provide flexibility in the choice of dosimetry workflow for validation, allowing variations in SPECT and WB imaging and timepoints. However the pharmacokinetic and absorbed dose ground truths are isotope and treatment specific. As such validation of a dosimetry system for the full range of MRT will require additional datasets to be produced. The use of Monte Carlo generated imaging with accompanying absorbed dose calculations has been shown to have potential to provide these data sets [32].

## 5 Conclusions

A methodology for production of a validation dataset for image based ^177^Lu dotatate dosimetry calculations has been presented. A 3D printed phantom has been designed, manufactured, and imaged to mimic a patient undergoing PRRT. A pharmacokinetic model was implemented and the dispensed activity concentration in each phantom compartment was in agreement with the pharmacokinetic model within measurement uncertainties at the acquisition time with body contours. SPECT and WB imaging of the phantom was performed at six clinically-relevant time points and the data has been made available in an online repository. The online database of SPECT projections, CT data and reconstructed data, with accompanying absorbed dose calculations, allows institutions and commercial dosimetry software developers to perform dosimetry on the phantom data and compare the results to a common ground truth. The data can also be used by individual institutions to investigate the effects of reconstruction parameters on quantitative imaging or to test partial volume correction algorithms on anthropomorphic phantom data. The STL files required to print a copy of the phantom have also been made available and any institution who would like to borrow the phantom is invited to contact the authors.

## CRediT authorship contribution statement

**Andrew P. Robinson:** Design of the 3D printed phantom, Data repository, Manuscript preparation, Coordinated the funding project. **Nick Calvert:** Design and production of the 3D printed phantom, Data collection and analysis, Manuscript preparation. **Jill Tipping:** Design and production of the 3D printed phantom, Data collection and analysis, Manuscript preparation. **Ana M. Denis-Bacelar:** Design of the 3D printed phantom, Manuscript preparation. **Kelley M. Ferreira:** Design of the 3D printed phantom, Manuscript preparation. **Michael Lassmann:** Design of the 3D printed phantom, Data collection methodology, Manuscript preparation. **Johannes Tran-Gia:** Design of the 3D printed phantom, Data collection methodology, Manuscript preparation.

## Declaration of Competing Interest

The authors declare that they have no known competing financial interests or personal relationships that could have appeared to influence the work reported in this paper.

## Appendix Collaboration members

**Manuel Bardiès** U1194 INSERM/ICM/Montpellier University(IRCM) and Regional Cancer Institute of Montpellier (ICM), Montpellier France

**Salvatore Berenato** Velindre Cancer Centre, Medical Physics,Cardiff, Wales, United Kingdom

**Ilias Bilas** National Physical Laboratory, Hampton Road, Teddington, United Kingdom

**Christophe Bobin** CEA, LIST, Laboratoire National Henri Becquerel, (LNE-LNHB), 91191, Gif-sur-Yvette Cedex, France

**Nicholas Calvert** Christie Medical Physics and Engineering (CMPE), The Christie NHS Foundation Trust, Manchester, United Kingdom

**Marco Capogni** ENEA, National Institute of Ionizing Radiation Metrology, Via Anguillarese 301, 00123 Rome, Italy

**Maxime Chauvin** CRCT, UMR 1037, INSERM, Université Toulouse III Paul Sabatier, Toulouse, France

**Sean Collins** National Physical Laboratory, Hampton Road, Teddington, United Kingdom

**Maurice Cox** National Physical Laboratory, Hampton Road, Teddington, United Kingdom

**Jérémie Dabin** Belgian Nuclear Research Centre (SCK CEN), Boeretang 200, 2400 Mol, Belgium

**Marco D’Arienzo** ENEA, National Institute of Ionizing Radiation Metrology, Via Anguillarese 301, 00123 Rome, Italy

**Ana M. Denis-Bacelar** National Physical Laboratory, Hampton Road, Teddington, United Kingdom

**Andrew J. Fenwick** National Physical Laboratory, Hampton Road, Teddington, and Cardiff University, Cardiff, United Kingdom

**Kelley M. Ferreira** National Physical Laboratory, Hampton Road, Teddington, United Kingdom

**Domenico Finocchiaro** Azienda Unità Sanitaria Locale di Reggio Emilia - IRCCS, Medical Physics Unit, Reggio Emilia, and Department of Physics and Astronomy, University of Bologna, Italy

**Federica Fioroni** Azienda Unità Sanitaria Locale di Reggio Emilia - IRCCS, Medical Physics Unit, Reggio Emilia, Italy

**Elisa Grassi** Azienda Unità Sanitaria Locale di Reggio Emilia - IRCCS, Medical Physics Unit, Reggio Emilia, Italy

**Aida Hallam** Radiation Physics and Protection, Churchill Hospital, Oxford University Hospitals NHS Foundation Trust, Oxford OX3 7LE, United Kingdom

**Warda Heetun** National Physical Laboratory, Hampton Road, Teddington, United Kingdom

**Stephanie Jewitt** Radiation Physics and Protection, Churchill Hospital, Oxford University Hospitals NHS Foundation Trust, Oxford OX3 7LE, United Kingdom

**Gunjan Kayal** CRCT, UMR 1037, INSERM, Université Toulouse III Paul Sabatier, Toulouse, France Belgian Nuclear Research Centre (SCK CEN), Boeretang 200, 2400 Mol, Belgium

**Maria Kotzassarlidou** Nuclear Medicine Dept., “THEAGENIO” Cancer Hospital, Thessaloniki, Greece

**Michael Lassmann** Department of Nuclear Medicine, University Hospital Würzburg, Würzburg, Germany

**Giuseppe Lorusso** National Physical Laboratory, Hampton Road, Teddington, United Kingdom

**Michael Ljungberg** Medical Radiation Physics, Lund, Lund University, Lund, Sweden

**Franz-Josef Maringer** BEV, Bundesamt für Eich- und Vermessungswesen, Arltgasse 35, 1160 Wien, Austria

**Daniel R. McGowan** Radiation Physics and Protection, Churchill Hospital, Oxford University Hospitals NHS Foundation Trust, Oxford OX3 7LE, and Department of Oncology, University of Oxford, Old Road Campus Research Building, Oxford, United Kingdom

**Darren Morgan** Radiation Physics and Protection, Churchill Hospital, Oxford University Hospitals NHS Foundation Trust, Oxford OX3 7LE, United Kingdom

**Andrew P. Robinson** National Physical Laboratory, Hampton Road, Teddington, and Christie Medical Physics and Engineering (CMPE), The Christie NHS Foundation Trust, Manchester, and The University of Manchester, Manchester, United Kingdom

**Nathaniel Scott** Radiation Physics and Protection, Churchill Hospital, Oxford University Hospitals NHS Foundation Trust, Oxford OX3 7LE, United Kingdom

**James Scuffham** Royal Surrey County Hospital, Guildford, UK and Department of Physics, University of Surrey, Guildford, United Kingdom and National Physical Laboratory, Hampton Road, Teddington, United Kingdom

**Vere Smyth** National Physical Laboratory, Hampton Road, Teddington, United Kingdom

**Jaroslav Šolc** Czech Metrology Institute, Okruzni 31, 638 00, Brno, Czech Republic

**Katarina Sjögreen Gleisner** Medical Radiation Physics, Lund, Lund University, Lund, Sweden katarina.

**Ludmila Štemberková** Czech Metrology Institute, Okruzni 31, 638 00, Brno, Czech Republic

**Lara Struelens** Belgian Nuclear Research Centre (SCK CEN), Boeretang 200, 2400 Mol, Belgium

**Jill Tipping** Christie Medical Physics and Engineering (CMPE), The Christie NHS Foundation Trust, Manchester, United Kingdom

**Johannes Tran-Gia** Department of Nuclear Medicine, University Hospital Würzburg, Würzburg, Germany

**Alex Vergara-Gil** CRCT, UMR 1037, INSERM, Université Toulouse III Paul Sabatier, Toulouse, France

**Jill Wevrett** Royal Surrey County Hospital, Guildford, and National Physical Laboratory, Hampton Road, Teddington, and Department of Physics, University of Surrey, Guildford, United Kingdom

**Hannah Wiedner** BEV, Bundesamt für Eich- und Vermessungswesen, Arltgasse 35, 1160 Wien, Austria
